# Effect of Nano-Montmorillonite on Osteoblast Differentiation, Mineral Density, and Osteoclast Differentiation in Bone Formation

**DOI:** 10.3390/nano10020230

**Published:** 2020-01-28

**Authors:** Gyeong-Ji Kim, Daniel Kim, Kwon-Jai Lee, Daeyoung Kim, Kang-Hyun Chung, Jeong Woo Choi, Jeung Hee An

**Affiliations:** 1 Department of Food and Nutrition, KC University, Seoul 07661, Korea; kgj8495@hanmail.net; 2 Department of Biomedical Engineering, Sogang University, Seoul 04107, Korea; 3 Advanced Geo-materials R&D Department, Pohang Branch, Korea Institute of Geoscience and Mineral Resources, Pohang 37559, Korea; daniel@kigam.re.kr; 4 Department of Advanced Materials Engineering, Daejeon University, Daejeon 34520, Korea; jmul@ssu.ac.kr; 5 Department of Nanomaterials Science and Engineering, University of Science and Technology, Daejeon 34113, Korea; daeyoung@kigam.re.kr; 6 Department of Food Science and Technology, Seoul National University of Science & Technology, Seoul 01811, Korea; carl@seoultech.ac.kr; 7 Department of Chemical and Biomolecular Engineering, Sogang University, Seoul 04107l, Korea

**Keywords:** nano-montmorillonite, bone formation, osteoblasts, osteoclasts, ovariectomy rats

## Abstract

Calcium-type montmorillonite, a phyllosilicate mineral, has diverse health benefits when introduced into the gastrointestinal tract or applied to the skin. However, the predominant use of this layered material has thus far been in traditional industries, despite its potential application in the pharmaceutical industry. We investigated the effects and mechanism of nano-montmorillonite (NM) on osteoblast and osteoclast differentiation in vivo and in vitro. We examined the osteogenic effects of NM with high calcium content (3.66 wt%) on alkaline phosphatase (ALP) activity, mineralization, bone microarchitecture, and expression level of osteoblast and osteoclast related genes in Ca-deficient ovariectomized (OVX) rats. Micro-computed tomography of OVX rats revealed that NM attenuated the low-Ca-associated changes in trabecular and cortical bone mineral density. It improved ALP activity and mineralization, as well as the expression of osteoblast and osteoclast differentiation associated genes. NM also activated the expression of runt-related transcription factor 2, osteocalcin, bone morphogenetic protein 2, and type 1 collagen via phosphorylated small mothers against decapentaplegic homolog 1/5/8 signaling. Further, NM repressed the expression of receptor activator for cathepsin K, nuclear factor kappa-B ligand and tartrate-resistant acid phosphatase. Therefore, NM inhibits osteoclastogenesis, stimulates osteoblastogenesis, and alleviates osteoporosis.

## 1. Introduction

Montmorillonite—a predominantly dioctahedral smectite—is a charged, layered silicate mineral and the main component of “bentonite” [1,2,3]. Bentonite is mainly included of montmorillonite and minor minerals, such as feldspar, albite, and quartz, depending on the geological environment [4]. Bentonite ore has thus far been widely used as drilling mud, animal feed additive, adsorbent, and soil improvement agent [5,6,7,8]. Recently, nearly pure montmorillonite purified from bentonite has been extensively studied for its use in cosmetics, natural therapies, and pharmaceuticals [9,10,11,12], owing to its specific properties such as high swelling pressure, high cation exchange capacity, and high adsorption ability [13,14,15]. In pharmaceutical applications, montmorillonite was found to have an active role in dermatological protection and anti-inflammation, and as an antidiarrheal and antacid [16,17,18,19]. Oral administration of montmorillonite as a dietary supplement showed protection against high-fat diet-induced dysbiosis of gut microbiota in mouse and prevention of obesity, by enhancing lipid excretion from the digestive tract in rats [20,21]. Recently, the effect of clay montmorillonite and sodium silicate was studied in human mesenchymal stem cells in osteogenic culture to confirm its role as a biomaterial [22]. The use of montmorillonite for bone therapy has also been reported, such as in engineered scaffolds with cytocompatibility, improving thermal stability, and increasing cellular proliferation rates [23,24].

Osteoporosis is a disease prevalent in postmenopausal women and is characterized by progressive loss of bone mineral density (BMD), suppressed bone strength, and enhanced fracture risk [25]. Deficiency of estrogen due to halted ovarian function is a pivotal role in the loss of bone mass in postmenopausal women [26]. Pathological factors for osteoporosis include imbalanced calcium (Ca) metabolism, decreased osteoblast activity, increased osteoclast function, and deficient estrogen production [27,28]. Generally, Ca has an important role in the human body, with various physiological functions. Ca is involved in structural support, muscle contraction, bone strength, blood clotting, and heartbeat regulation [29]. Ca intake induce to an increase in BMD and can suppress the risk of fracture and osteoporosis [30]. Clinical research has many interested in fracture prevention and treatment for the improvement of BMD [31]. Previous studies have investigated the use of Ca supplements such as calcium lactate, calcium citrate, and calcium carbonate extracted from foods [32,33,34]. Anke et al. (1992) showed that goats fed a ration of bentonite had decreased Ca, phosphorus, and sodium content in bone [35]. Dembinski et al. (1985) found that supplementation of bentonite at 2000 mg/kg body weight in hybrid goats decreased Ca deposition in bone [36]. However, studies on the bone formation mechanism of montmorillonite have not been reported.

The objective of this study was to observe the in vitro and in vivo effects of nano-montmorillonite (NM) on bone remodeling by osteoblastogenesis and osteoclastogenesis. We demonstrate here, for the first time, by modulating osteoblast and osteoclast-specific genes, transcription factors, and signaling molecules, that NM promotes osteoblast differentiation via bone morphogenetic protein 2 (BMP-2)/phosphorylated small mothers against decapentaplegic homolog 1/5/8 (p-SMAD 1/5/8)/runt-related transcription factor 2 (RUNX2) expression and suppresses osteoclast differentiation via tartrate-resistant acid phosphatase (TRAP)/receptor activator of nuclear factor kappa-B (RANK). Our results suggest that NM could open a new field of study of mineral materials and the application of these materials in the treatment of osteoporosis.

## 2. Materials and Methods

### 2.1. Preparation of NM

Natural bentonite was collected from the Gampo-40 mining site located in Gyeongju, South Korea. Montmorillonite was purified from fresh bentonite by a sedimentation and segregation processes for particle sizes ≤5 μm, followed by oven drying at 80 °C overnight. To prepare the NM suspension used in this study, purified montmorillonite (6.2 mg) was dispersed in deionized water (1 mL) by sonication with glass beads for 3 h.

### 2.2. Powder X-Ray Diffraction (XRD)

The powder XRD pattern was determined using a Rigaku Mini Flex 600 diffractometer with Cu Kα radiation (λ = 1.5406 Å) at 40 kV and 15 mA. Data were collected over a 2θ range of 2° to 70°, with a step size of 0.02°. Indexing of the obtained XRD patterns was analyzed by the Crystallographica Search-Match (CSM) software (Oxford Cryosystems, Oxford, UK).

### 2.3. Scanning Electron Microscopy (Sem) and Energy Dispersive X-Ray Spectroscopy (EDS)

Purified montmorillonite was coated with palladium, and the morphology and atomic ratio of the sample were investigated by SEM (Model EVO LS25, ZIESS), operating at 10 kV, equipped with an energy dispersive X-ray spectrometer (EDS, NORAN SYSTEM7, Thermo Scientific).

### 2.4. Particle Size Analysis

The particle size and distribution of the NM suspension were measured using a Microtrac S3500 Bluewave Laser Diffraction instrument.

### 2.5. Cell Culture

Osteoblast-like cell (MG-63) and osteoclast cell (RAW 264.7) (Korean Cell Bank, Seoul, Korea) were grown in Roswell Park Memorial Institute (RPMI) medium or Dulbecco’s modified Eagle’s medium (DMEM) supplemented with fetal bovine serum (FBS, 10%; Hyclone, Logan, UT, USA) and penicillin-streptomycin (1%; GIBCO, Grand Island, NY, USA) in a CO_2_ (5%) incubator at 37 °C.

### 2.6. Cell Viability Assay

MG-63 (5 × 10^4^ cells/mL) and RAW 264.7 (5 × 10^4^ cells/mL) cells were differentiated in a 96-well plate. The cell viability was determined by 3-(4,5-dimethylthiazol-2-yl)-2,5-diphenyltetrazolium bromide (MTT; Promega, Madison, WI, USA) analysis. The cells were treated with various concentrations (250–1000 μg/mL) of NM for 24 h. After 24 h of exposure, the 20 µL of MTT solution (5 mg/mL) was added. After 4 h, the formazan crystals had formed and dissolved in 200 µL of dimethyl sulfoxide (DMSO). Then, the absorbance of the resulting solution was evaluated based on the optical density values measured at 540 nm using a microplate reader (Biochrom Asys, Cambridge, UK).

### 2.7. Analysis of Alkaline Phosphatase (ALP) Activity

The ALP activity was analyzed as the rate of *p*-nitrophenyl phosphate (p-NPP) hydrolysis. MG-63 cells (5 × 10^4^ cells/mL) were seeded in 96-well plates. For induction and cell differentiation, cells were treated with β-glycerol phosphate (10 mM) and ascorbic acid (50 mg/mL) and the indicated concentrations of NM for 7 days. The cells were lysed in Triton X-100 (0.1%), and incubated with p-NPP at 37 °C for 60 min. Then, the ALP activity was measured at 405 nm using a microplate reader.

### 2.8. Bone Mineralization Analysis Using Alizarin Red S Staining

The mineralization was determined after 14 days of treatment with differentiation medium and NM. For Alzarin red S staining, the MG-63 cells were fixed in 70% ethanol for 1 h, incubated with alizarin red S (40 mM) in deionized water (pH 4.2) for 15 min. Then, the cells were washed the PBS, and then de-stained for 15 min with cetylpyridinium chloride (10% w/v) in sodium phosphate (10 mM, pH 7.0). The absorbance was measured at 550 nm using a microplate reader.

### 2.9. Tartrate-Resistant Acid Phosphatase (Trap) Activity and Staining

RAW 264.7 cells (5 × 10^4^ cells/mL) were seeded in 96-well plates. Five days after stimulating the cells with RANK ligand (RANKL, 50 ng/mL), the cells were washed with PBS. To measure TRAP activity, the cells were fixed in 3.5% formaldehyde for 10 min. Then, the cells rinsed with distilled water, and incubated in citrate buffer (50 mM, pH 4.5) containing sodium tartrate (10 mM) and p-NPP (6 mM). After 1 h, the mixtures solution was transferred to new 96-well plates containing an equal volume of NaOH (0.1 N). The absorbance was measured at 405 nm in a microplate reader. The cells were stained for TRAP by a leukocyte acid phosphatase assay kit (Sigma), following the manufacturer.

### 2.10. Animals and Diet

All animal experiments were performed with the approval from the Institutional Animal Care and Use Committee at Konkuk University (IACUC approval number KU 15133). Female Wistar rats weighing 117 ± 7 g and aged 5 weeks (Doo Yeol Biotech, Seoul, Korea) were used in this study. The animals were maintained in a room at 22 °C with a 12 h light-dark cycle. After acclimatization for one week, the rats were anesthetized with 2% isoflurane, and their ovaries were removed bilaterally. Rats underwent a sham operation (n = 12), by exposing ovaries without excision, or ovariectomy (OVX, n = 36), by ligating and excising the ovaries. After a 2-week acclimation period, the sham and normal diet groups were fed a normal (0.6% Ca) diet (TD.97191), while the other groups were fed a low-Ca (0.01%) diet (TD.95027). The animals were randomly divided into four groups (12 rats per group): (1) Sham (normal diet); (2) low-Ca (OVX + low-Ca); (3) normal diet (OVX + normal diet); and (4) NM (OVX + low-Ca + 50 mg/kg NM) (Table S1). The dietary supplement composition is shown in the Supplementary Material (Table S1). The food intake was monitored daily, and the rats were weighed once weekly. The rats were dosed 50 mg NM/kg body weight via drinking water ad libitum. At the end of the 8-week feeding period, the rats were dissected. Blood samples were collected from the heart while the animal was lightly anaesthetized, and serum was separated by centrifugation (848 g for 30 min) and then stored at −80 °C prior to the biochemical analyses. Tibial bones were dissected and stored at −80 °C.

### 2.11. Biochemical Analyses

Serum alanine aminotransferase (ALT), aspartate aminotransferase (AST) levels, total cholesterol and the high-density lipoprotein (HDL)-cholesterol were measured using commercial kits (Asan Pharm Co. Ltd., Seoul, Korea). Tibial Ca content was measured as follows: Subsequent to the densitometry measurements, the tibias were ashed in a muffle furnace at 600 °C for 4 h. The ash was weighed and then dissolved in 4 N HCl prior to Ca determination by atomic absorption spectroscopy (Varian AA-20, Varian Analytical Instruments, Walnut Creek, USA) and phosphorus analysis by visible spectroscopy (Varian Cary One), using AOAC procedures.

### 2.12. Bone Strength Test

The breaking force of the tibia was measured using an A/WEG wedge fracture probe (Stable Micro Systems, Godalming, UK). The wedge was fractured by a downward motion (3 mm/s) of a 30 mm-wide steel blade. The maximum force (N) applied to break the wedge was used to quantify bone firmness.

### 2.13. Measurement of Tibial Bone Ca Content

The Ca content of the tibias was quantified by inductively coupled plasma mass spectrometry (ICP-MS, HP-4500; Hewlett-Packard, Avondale, PA, USA), using a microwave digestion system (Multiwave 3000; Anton Paar, Graz, Austria). All tests were performed following AOAC procedures.

### 2.14. Micro-Computerized Tomography (Micro-CT)

Tibial morphometric parameters were measured in the distal tibia using a high-resolution cone-beam micro-CT system (Inveon PET; Siemens Medical Solutions, Knoxville, TN, USA). The bone surface area/bone volume (BSA/BV), bone volume/total volume (BV/TV), trabecular separation (Tb.Sp), trabecular thickness (Tb.Th), and trabecular number (Tb.N) were calculated from 3D measurements of the trabecular bone mass and its distribution. The cortical wall thickness (Ct.Th) was calculated from 3D measurements of cortical bone mass and its distribution, according to standard procedures. Scans were performed using 80 kV of applied voltage with a 1 mm aluminum filter. All cross-sections contained 2048 × 2048 pixels, with an isotropic voxel size of 9.31 µm. Data analysis was conducted using the Inveon Acquisition Workplace software (Siemens).

### 2.15. Histological Evaluation

The tibias were fixed in 10% neutral-buffered formalin for 2 days at 40 °C. Decalcification was achieved by immersion in 10% ethylenediaminetetraacetic acid (EDTA, pH 7.4), which was replaced daily for 20 days, with stirring, at room temperature. The bones were washed in tap water for 4 h, the tibias were embedded in paraffin, and longitudinal 4 μm sections were cut using a microtome. The sections were mounted, stained using hematoxylin and eosin (H&E), and observed by light microscopy at a magnification of 100×.

### 2.16. Von Kossa Staining

Formalin- and ethanol-fixed tibia samples were incubated with 1% silver nitrate. Then, tibia samples placed under UV light at 10 min, rinsed in distilled water, and unreacted with 5% sodium thiosulfate for 5 min. The samples were stained with 0.1% Nuclear Fast Red solution for 5 min. Then, the samples were dehydrated with grade ethanol and mounted using DPX mounting medium.

### 2.17. Reverse Transcriptase PCR (RT-PCR)

Rat tibia RNA was isolated at the TRIzol soultion (Invitrogen, Carlsbad, CA, USA). Aliquots (1 μg) of total RNA were reverse transcribed using SuperScript III Reverse Transcriptase (Invitrogen). The resultant cDNA was used to determine the tibial mRNA expression levels of BMP-2, cathepsin K, COL-1, osteocalcin, RANK, RUNX2, TRAP, and Wnt3a using Taq DNA polymerase (KAPA Biosystems, London, UK). Glyceraldehyde 3-phosphate dehydrogenase (GAPDH) was used as an internal control. The PCR primers used are listed in Table S2. The following thermocycling conditions were used: 95 °C for 3 min; 30–40 cycles at 95 °C for 30 s and 50–60 °C for 30 s (the number of cycles and annealing temperatures were optimized for each primer pair); and 72 °C for 10 min. The PCR products were separated by agarose (1.2%)/ethidium bromide gel electrophoresis and photographed.

### 2.18. Immunohistochemical Analysis

Sections (4 μm) of the decalcified and paraffin-embedded tibias were mounted onto 3-aminopropyltriethoxysilane (APES)-coated slides, deparaffinized in xylene, rehydrated using ethanol, and rinsed in PBS. Endogenous peroxidase activity was quenched by incubation in hydrogen peroxide (0.3%) for 30 min. The sections were incubated with goat serum (10%) for 30 min before overnight incubation at 4 °C with the appropriate specific primary antibodies raised against BMP-2, RUNX2, Wnt3a, osteocalcin, or COL-1 (Abcam, Cambridge, MA, UK). The sections were then incubated with either biotinylated goat anti-rabbit IgG (H+L) horseradish peroxidase (HRP) conjugate (Zymax, San Francisco, CA, USA) or goat anti-mouse IgG (H+L) HRP conjugate (Zymax). The sections were then stained with 3,3′-diaminobenzidine (DAB Substrate Kit; Vector Laboratories, CA, USA) and counterstained with hematoxylin. Negative controls were incubated with normal goat IgG instead of the primary antibody. The specimens were examined using an Eclipse TS100 microscope (Nikon, Tokyo, Japan) at 200× magnification, and the images were analyzed using the Optiview 3.7 software (Korea Lab Tech, Sungnam, Korea).

### 2.19. Western Blot Analyses

Tibias were homogenized in RIPA lysis buffer including protease inhibitor (Roche, Mannheim, Germany) and centrifuged at 10,000× *g* for 10 min at 4 °C. The total protein levels were determined using a Bio-Rad Protein Assay Kit. The proteins were subjected to SDS-PAGE and transferred onto Immobilon-P transfer membranes, which were blocked with bovine serum albumin (5%) prior to incubation with specific primary antibodies raised against BMP-2, Wnt3a, RUNX2, or COL-1 (Abcam); p-SMAD 1/5/8 (Santa Cruz, Texas, USA); or p-ERK, p-p38, p-JNK, or β-actin (Cell Signaling Technology, Danvers, MA, USA). The membranes were then incubated with the appropriate secondary antibodies, either goat anti-rabbit IgG H&L (HRP) (Abcam) or goat anti-mouse IgG H&L (HRP) (Abcam). The antigen-antibody complexes were visualized using enhanced chemiluminescence. Densitometric analysis was performed using a C-DiGit Blot Scanner (Li-COR Biosciences, Lincoln, NE, USA).

### 2.20. Statistical Analyses

Data are presented as the mean ± SD of triplicate experiments. Statistical analyses were conducted using the Statistical Package for the Social Sciences (SPSS) version 18.0 (SPSS Inc., Chicago, IL, USA). The groups were compared using one-way ANOVA followed by Duncan’s multiple range tests, and *p*-values < 0.05 were considered statistically significant.

## 3. Results

### 3.1. Characteristics of Montmorillonite

**Figure 1:**
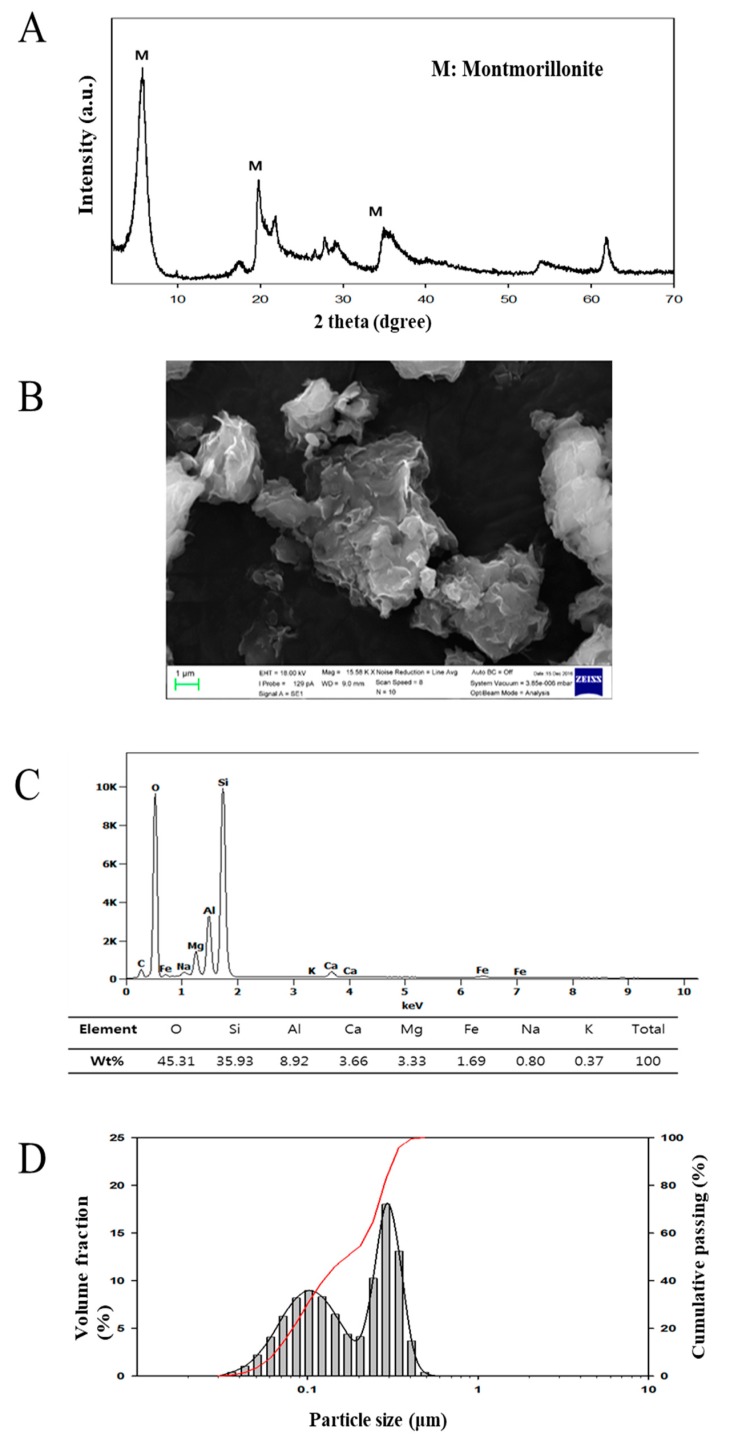
(**A**) X-ray powder diffraction (XRD) pattern, (**B**) scanning electron microscopy (SEM) image (magnified 15.58K-fold), (**C**) energy-dispersive X-ray spectroscopy (EDS) spectrum of purified montmorillonite, and (**D**) particle-size distribution of the nano-montmorillonite suspension.

Figure 1A shows the XRD pattern of the purified montmorillonite. The primary XRD peaks were around 2*θ* = 5.6°, 19.8, and 34.9°. These peaks confirmed a typical montmorillonite in the dioctahedral montmorillonite group [1,37]. The distance between the two-unit montmorillonite sheets from the powder sample was measured by the PDXL software. The calculated distance is 15.6 Å, which agrees with previously reported results for Ca-type montmorillonites [14,38,39]. The shape of the montmorillonite is described in Figure 1B using an SEM image. The observed size range of the particles was approximately 1 to 7 μm. The irregular size and morphology of the sample appears to be due to aggregation of montmorillonite particles or inter-particle contact during oven drying at 353 K. As determined by EDS and shown in Figure 1C, the quality and quantity index ratios of the peak areas were obtained from the EDS fingerprint spectra. The predominant elements in the montmorillonite sample were Si, Al, Fe, Mg, and Ca, in varying levels. The montmorillonite had high Ca (3.66 wt%) and low Na (0.80 wt%) content. The particle size distribution (PSD) of NM is summarized in Figure 1D. The sample PSD ranged from 65 to 338 nm. The size analysis results showed a bimodal distribution that centered at 87 and 264 nm, with an average of 179 nm. This bimodal PSD pattern is similar to the NM PSD graph described previously [40,41]. The average size is smaller than other NMs reported, which have average bimodal sizes of 280 nm in diameter [42]. In conclusion, the results confirmed that the sample was, indeed, a natural Ca-type NM.

### 3.2. NM Activates Alp and Mineralization in Osteoblasts

**Figure 2:**
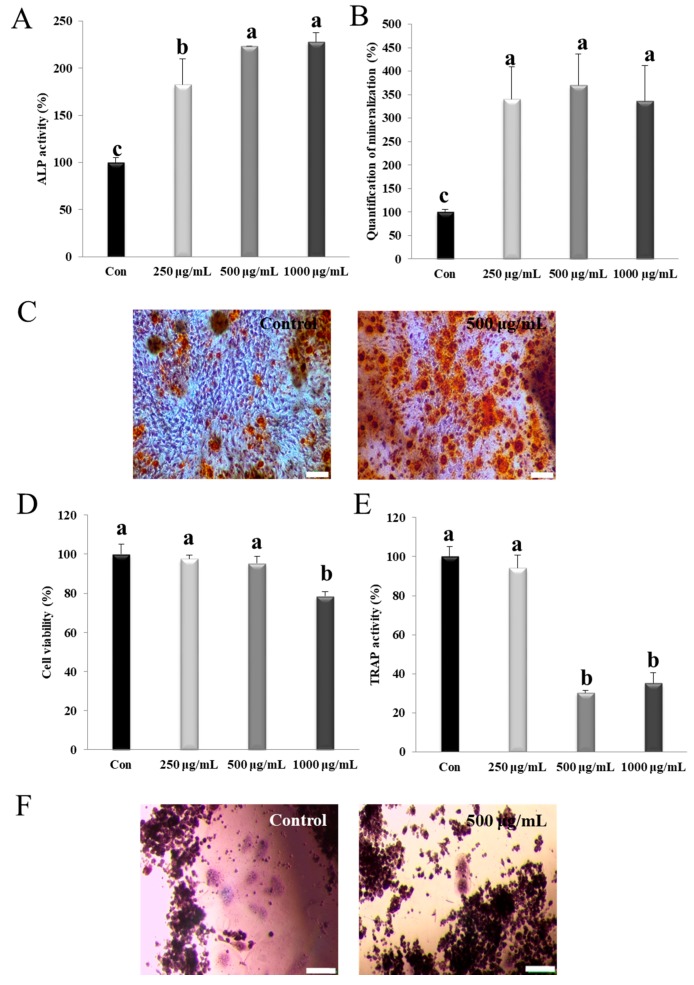
Effect of nano-montmorillonite (NM) on alkaline phosphatase (ALP) activity and mineralization during differentiation in MG-63 cells. (**A**) Cells were treated with the indicated NM concentrations for 24 h before measuring the cell viability and for 96 h before measuring the ALP activity, as described in Materials and Methods. Scale bar: 20 μm. (**B**,**C**) Cells were treated as indicated for 14 days before staining the mineralized matrix with alizarin red S and quantifying the mineralization as described in Materials and Methods. Effect of NM on RAW 264.7 cell viability (**D**), tartrate-resistant acid phosphatase (TRAP) activity (**E**), and TRAP staining (**F**, control and NM 500 μg/mL) during differentiation. Scale bar: 20 μm. Results are expressed as the mean ± SD. Values not sharing a common superscript (**a**–**c**) differed significantly (Duncan’s multiple range test) (*p* < 0.05).

NM did not exhibit any significant effect on cell viability for 72 h at the concentrations used in this study. Osteoblasts produce ALP in association with mineralization and matrix maturation [43]. As shown in Figure 2A, the NM-treated group showed significantly enhanced ALP activity, and the effect was dose-dependent from 250–1000 μg/mL (Figure 2A) (*p* < 0.05). The degree of mineralization in the NM-treated group was observed using alizarin red S counterstaining and quantified by Ca deposition analysis. The matrix mineralization of NM treated group (350%) was the higher than control group at 500 μg/mL concentration. The mineralization did not show significant in concentrations of NM (Figure 2B). The NM-treated cells demonstrated redder color than the control cells, indicating the promotion of bone mineralization (Figure 2C). Thus, NM effectively enhanced osteoblast maturation, by increasing bone mineralization.

### 3.3. NM Suppresses Osteoclast Differentiation in Osteoclast Precursor Cells

In prior to evaluate inhibitory effect of NM in osteoclast differentiation, cytotoxicity assay was performed in vitro. Thus, the cell was treated with three different concentrations of NM (250, 500, and 1000 μg/mL) to determine the concentrations that inhibited osteoclast precursor cell proliferation. NM concentration under 500 μg/mL did not show significant cytotoxicity in osteoclast precursor cells (Figure 2D). As shown in Figure 2E, the exposure to 1000 μg/mL NM significantly inhibited TRAP activity in multinucleated TRAP-positive RAW 264.7 cells. NM suppressed TRAP activity to 30.4% of the control value. As shown in Figure 2F, NM-treated RAW 264.7 cells exhibited greatly repressed TRAP-positive multinucleated cells, compared to that in the NM-untreated cells. Our results suggest that osteoclastogenesis was significantly decreased in RAW 264.7 cells treated to NM. This indicates that NM has the potential to suppress bone resorption during osteoporosis.

### 3.4. NM Enhances Expression of Osteoblast Differentiation Marker Genes via P38

**Figure 3:**
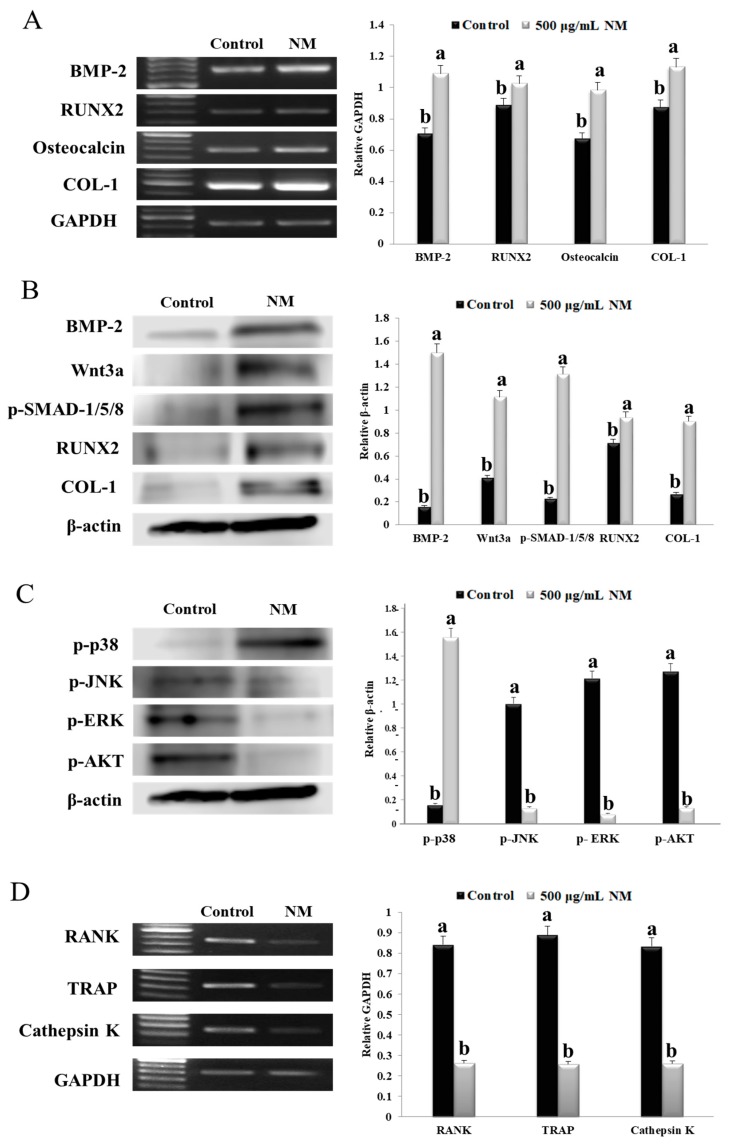
MG-63 osteoblast and RAW 264.7 osteoclast mRNA and protein expression levels of molecules involved in bone metabolism and signaling. (**A**,**B**) The cells were differentiated for 4 days in differentiation medium prior to reverse transcription PCR (RT-PCR) analysis of bone morphogenetic protein-2 (BMP-2), runt related transcription factor-2 (RUNX2), osteocalcin, and type 1 collagen (COL-1); mRNA levels and protein levels. (**C**) Levels of phosphorylated extracellular signal-regulated kinase (p-ERK), p-p38, phosphorylated serine/threonine kinase (p-AKT), and phosphorylated c-Jun N-terminal kinase (p-JNK) were determined by Western blot. (**D**) mRNA expression of receptor activator of nuclear factor kappa-B (RANK), tartrate resistant acid phosphatase (TRAP), and cathepsin K. The relative expression was quantified using ImageJ and calculated relative to glyceraldehyde 3-phosphate dehydrogenase (GAPDH) and β-actin. Results are expressed as the mean ± SD. Values not sharing a common superscript (**a**,**b**) differed significantly (Duncan’s multiple range test) (*p* < 0.05).

Figure 3A shows the mRNA expression levels of osteoblast differentiation-associated genes in MG-63 cells with NM treated for 3 days. Four genes involved in bone metabolism were analyzed: RUNX2, osteocalcin, BMP-2, and collagenase-1 (COL-1). The mRNA expression level of BMP-2 and RUNX2 increased by 1.53- and 1.15-fold in the NM-treated group compared to that in the control cell. In addition, the osteocalcin and COL-1 mRNA expression levels were 1.45- and 1.29-fold higher in the NM-treated cell than in the control group. As shown in Figure 3B, we estimated the effect of NM treated group on the bone metabolism indicators RUNX2, BMP-2, COL-1, Wnt3a and p-SMAD 1/5/8 in MG-63 cells. The protein expression levels were significantly increased for RUNX2, BMP-2, COL-1, Wnt3a and p-SMAD 1/5/8 in the NM-treated cells compared to the control cells. The BMP-2 and Wnt3a protein expression levels increased by 9.39- and 2.72-fold, respectively, in the NM-treated cells compared to the control cells. In addition, the p-SMAD 1/5/8, RUNX2, and COL-1 protein expression levels in the NM-treated group increased by 5.74-, 1.31-, and 3.36-fold, respectively, compared to that in the control cells. These findings suggested that BMP2-dependent p-SMAD 1/5/8/RUNX2/osteocalcin/COL-1 signaling was increased in cultured MG-63 cells exposed to NM, leading to osteoblast differentiation.

As shown in Figure 3C, the activation level of p38 was significantly increased (9.57-fold) in the cells treated with NM compared to that in the control cells. The NM-treated cells showed significantly reduced protein levels of p-AKT, p-JNK, and p-ERK compared to the control cells. These data suggested that p38 signaling was activated in cells treated with NM.

### 3.5. NM Inhibits Osteoclast Formation Genes

Expression level of cathepsin K, TRAP, and RANKL were significantly decreased in the NM-treated group compared to the control group (Figure 3D). In addition, the TRAP expression level in the NM-treated cells was 4.78-fold lower than those in the control group (Figure 3D). These results suggested that NM suppressed mRNA expression level of osteoclastogenesis related genes in vitro.

### 3.6. Food Efficiency Ratio

The body weight gain, food intake, and food efficiency ratio did not show significant differences among the experimental groups, as shown in the Supplementary Material (Table S3).

### 3.7. NM Improves High-Density Lipoprotein (HDL)-Cholesterol Lipid Metabolism and Prevents Atherosclerosis

There were no significant differences in the serum levels of ALT or AST between the different study groups, as shown in the Supplementary Material (Figure S1A). Total serum cholesterol was significantly higher in the NM-treated rats than in the other groups (Figure S1B). Especially, the level of HDL-cholesterol in the NM group increased by 19.7% compared to that in the low-Ca group. Also, HDL-cholesterol in the NM group was higher than in the normal diet group. These data imply that NM improves lipid metabolism as evidenced by the increased HDL-cholesterol level. The serum estrogen concentration reduced in the low-Ca group compared to the other groups (Figure S1C). Interestingly, the NM group showed an increased serum estrogen level (1.38-fold) compared to the low-Ca group.

### 3.8. NM Increases Tibial Ca Content and Bone Strength

**Figure 4:**
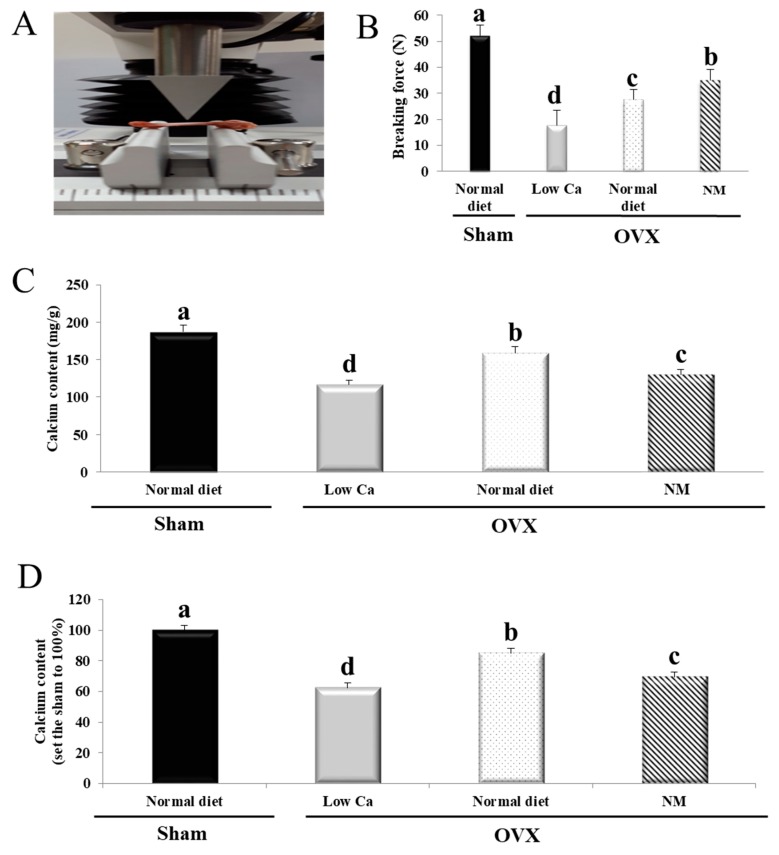
Breaking force graph for the rat tibia. (**A**) Details of the breaking force measurement by texture analysis. (**B**) Breaking force required for rat tibias. (**C**) Tibial Ca levels in the indicated rats. (**D**) The value of the tibial Ca levels when sham was set to 100%. Results are expressed as the mean ± SD. Values not sharing a common superscript (**a**–**c**) differed significantly (Duncan’s multiple range test) (*p* < 0.05).

To investigate the effects of NM-containing diets on bone strength, we analyzed a breaking force test using a texture analyzer (Figure 4A). The breaking force required in the NM group was 17.23% higher than in the low-Ca group (Figure 4B). In addition, the breaking force of the NM group (35.02 N) increased compared to the normal diet group (27.61 N). Tibial Ca levels were higher in the normal diet group than in the NM group (Figure 4C). However, the NM group had greatly increased Ca levels than the low-Ca group. The NM group had 33.7% more tibial Ca than the low-Ca group, which showed no significant difference from the sham group (Figure 4D). Thus, the NM diet increased the tibial bone strength and the concentration of Ca in bone, compared to the low-Ca group.

### 3.9. NM Improves BMD and Bone Microarchitecture

**Figure 5:**
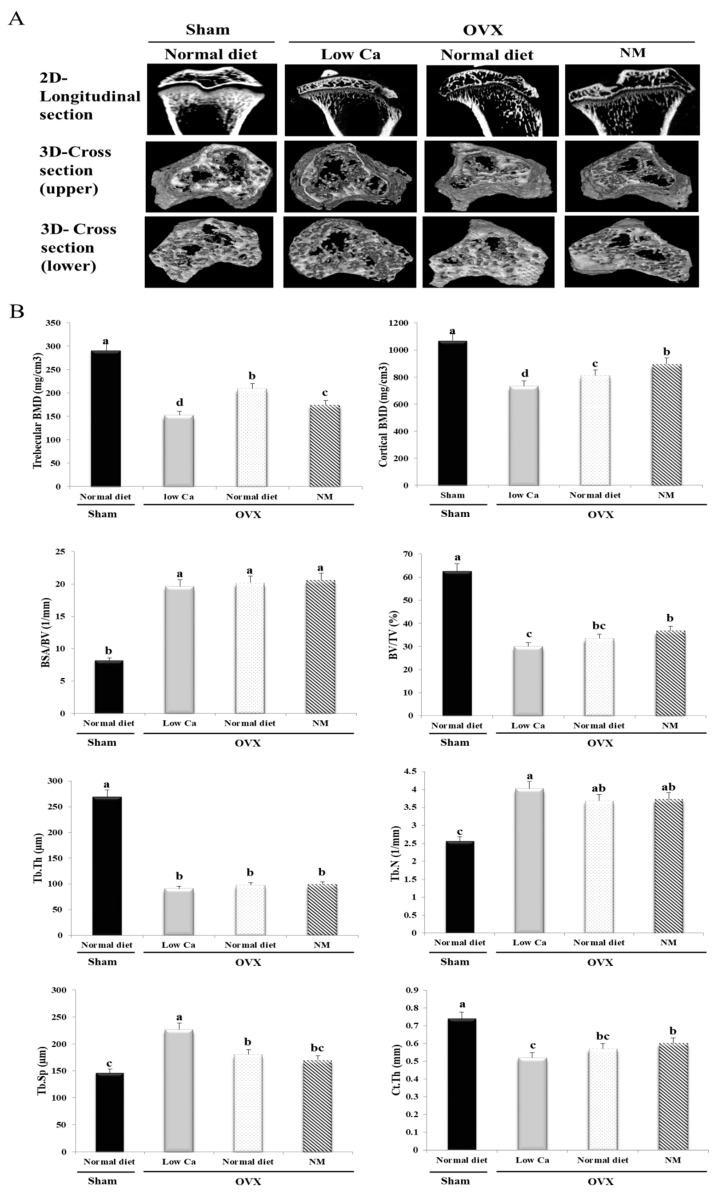
Tibial bone microstructure of ovariectomized (OVX) rats. (**A**) Longitudinal and cross-sections of trabeculae. (**B**) Trabecular bone and cortical bone analysis. Each value is expressed as the mean ± SD. Values not sharing a common superscript (a-c) differed significantly (Duncan’s multiple range test) (*p* < 0.05).

Ex vivo micro-CT was performed 8 weeks after OVX or sham surgery for all animals in the study; the tibial architecture was evaluated using the maximum intensity projection images (Figure 5A). Our study analyzed the trabecular and cortical compartments of the tibia, using measurements and images of longitudinal and cross-sections that indicated the location slab of the trabeculae. After 8 weeks, rats fed a low-Ca diet showed an overall loss of the tibia, indicated by an increase in bone spacing and decrease in the bone mass, bone marrow, and thickness (Figure 5A). The longitudinal sections of the tibias from the low-Ca group show central spaces in the trabeculae (Figure 5A). The low-Ca group shows large spaces within the marrow in the upper and lower tibial cross-sections. In contrast, the longitudinal and cross-sectional analyses of the sham and NM groups observed that the bone marrow space was more occupied in the NM group than in the low-Ca group. The sham and NM groups distributed bone structures in a relatively uniform fashion, forming a well-connected network (Figure 5A).

Micro-CT analysis was used to calculate trabecular BMD, cortical BMD, BV/TV, BSA/BV, Tb.Th, Tb.N, Tb.Sp, and Ct.Th (Figure 5B). The low-Ca feed decreased trabecular BMD, cortical BMD, BV/TV, Tb.Th, and Ct.Th. However, the low-Ca feed increased BSA/BV, Tb.N, and Tb.Sp. In addition, NM increased trabecular BMD, cortical BMD, BV/TV, and Ct.Th by 7.56%, 15.15%, 10.80%, and 10.81%, respectively, compared to the low-Ca group. Furthermore, cortical BMD, BV/TV, and Ct.Th increased in the NM group, compared to the normal diet group. Tb.N and Tb.Sp decreased by 11.98% and 39.36%, respectively, compared to the low-Ca group. However, BSA/BV and Tb.Th were not significantly different in the low-Ca and NM groups (Figure 5B). These data indicated that dietary supplementation with NM attenuated the loss in integrity of the trabeculae in OVX rats. These effects were confirmed by histological analyses using H&E staining.

### 3.10. NM Increases Trabecular Connections

**Figure 6:**
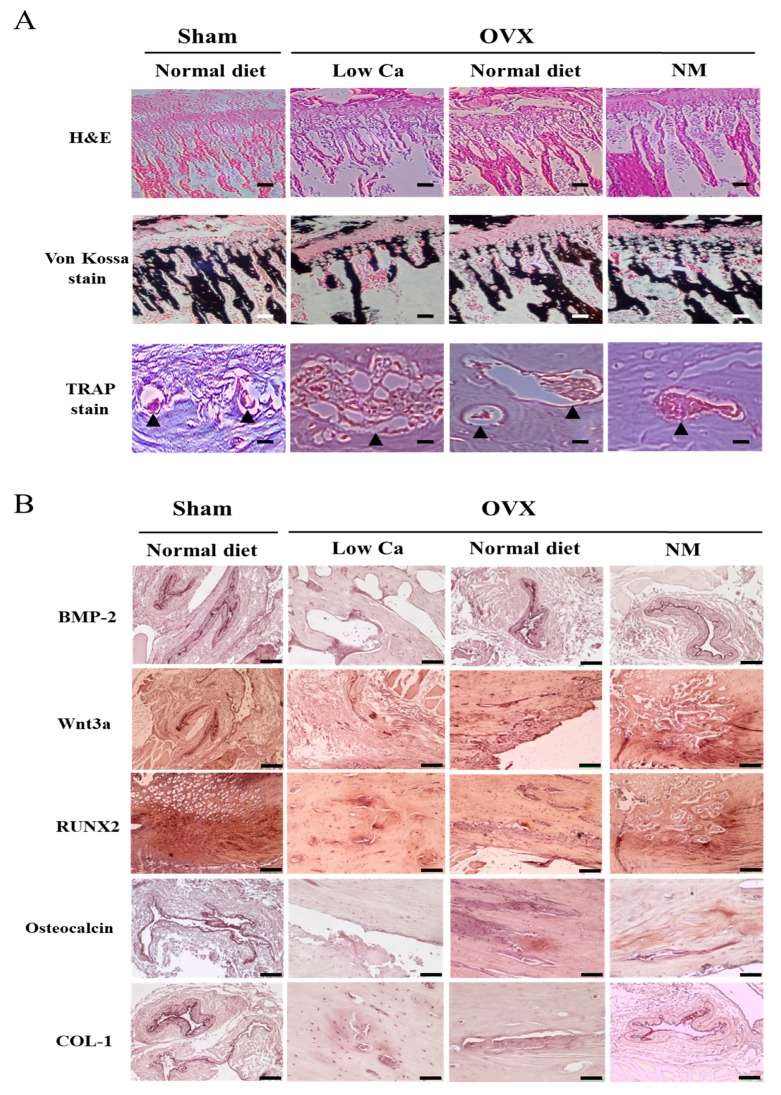
Histomorphology of the tibia of ovariectomized (OVX) rats. (**A**) Hematoxylin and Eosin (H&E) stain, von Kossa stain, and tartrate-resistant acid phosphatase (TRAP) stain of the tibia. Black triangles indicate osteoclasts. Scale bar: 20 μm (**B**) Immunohistochemical staining image of bone morphogenetic protein-2 (BMP-2), Wnt family member 3a (Wnt3a), runt-related transcription factor-2 (RUNX2), osteocalcin, and type 1 collagen (COL-1) in OVX rats. Scale bar: 100 μm.

To gain insight into the mechanism of the enhanced bone formation in OVX rats, we performed histomorphometric analysis. The bone trabeculae of the rats in the low-Ca group were arranged randomly, were thinner, or had disappeared altogether, while the remaining connections were incomplete (Figure 6A). Trabecular continuity was not optimal, and there were apparent signs of resorption. However, the arrangement of the trabeculae in the NM group showed that a greater number connected to form a bone network, and in a more orderly arrangement than what was observed in the low-Ca group (Figure 6A). The analysis indicated that osteoid formation improved with NM supplementation, compared to the low-Ca group. By von Kossa staining, differences might not be observed between the sham, normal diet, and NM groups.

We showed by H&E staining that the osteoclast activity was increased in the endochondral zone and epiphysis of the rates in the low-Ca group, as evidenced by the increased staining of the osteoclast-specific marker TRAP (Figure 6A). These findings indicate that NM dietary supplementation improves trabecular structure and continuity.

Immunohistochemical staining was conducted to detect the osteogenesis-related proteins BMP-2, RUNX2, Wnt3a, osteocalcin, and COL-1. For BMP-2, the connective bone tissue surrounding the trabeculae showed mild staining in the low-Ca group, and a small trend towards increased staining was observed in the sham and NM groups (Figure 6B). For Wnt3a, the low-Ca group showed a mild staining pattern in the bone tissue surrounding the trabeculae, while intense staining was showed in the NM group. Additionally, the NM group showed moderate RUNX2 staining in the connective tissue surrounding the trabeculae. The low-Ca group showed mild staining for osteocalcin and COL-1, while the sham and NM groups showed more intense staining. Furthermore, Wnt3a, RUNX2, and COL-1 staining was more intense in the NM group compared to the normal diet group. These results demonstrated that NM supplementation increased the bone levels of the osteogenesis-related BMP-2, RUNX2, Wnt3a, osteocalcin, and COL-1 proteins.

### 3.11. NM Promotes in Vivo mRNA and Protein Expression of Genes Involved in Osteoblastogenesis

**Figure 7:**
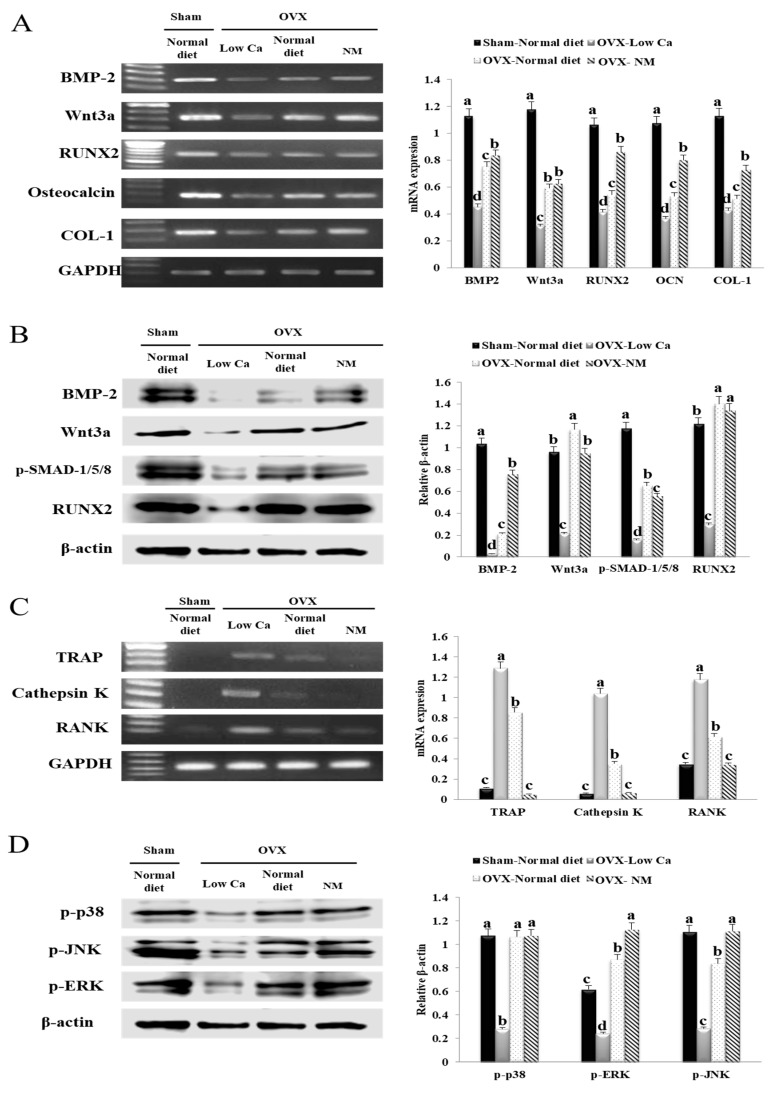
In vivo gene expression and histomorphology of the tibia of ovariectomized (OVX) rats. (**A**) mRNA expression levels of osteoblast differentiation factors: bone morphogenetic protein-2 (BMP-2), Wnt family member 3a (Wnt3a), runt-related transcription factor-2 (RUNX2), osteocalcin, and type-1 collagen (COL-1). (**B**) Protein expression levels of osteoblast differentiation factors: BMP-2, Wnt3a, phosphorylated small mothers against decapentaplegic homolog 1/5/8 (p-SMAD 1/5/8), and RUNX2. (**C**) Expression levels of phosphorylated extracellular signal-regulated kinase (p-ERK), p-p38, phosphorylated serine/threonine kinase (p-AKT), and phosphorylated c-Jun N-terminal kinase (p-JNK). (**D**) mRNA expression levels of receptor activator of nuclear factor kappa-B (RANK), tartrate resistant acid phosphatase (TRAP), and cathepsin K in vivo. Each value is expressed as the mean ± SD. Values not sharing a common superscript (**a**–**d**) differed significantly (Duncan’s multiple range test) (*p* < 0.05).

The tibial mRNA and protein expression levels are shown in Figure 7A,B. The mRNA and protein levels of the bone metabolism indicators BMP-2, Wnt3a, RUNX2, osteocalcin, and COL-1, were significantly upregulated in the NM group compared to the low-Ca group. In the NM group, the BMP-2 and Wnt3a mRNA levels were 1.84- and 2.01-fold higher, respectively, than in the low-Ca group. Also, the mRNA levels of RUNX2, osteocalcin, and COL-1 in the NM group increased by 2.08-, 2.19-, and 1.71-fold, respectively, compared to the low-Ca group. Furthermore, the NM group significantly increased the expression of RUNX2, osteocalcin, and COL-1. The increased expression of RUNX2, osteocalcin, and COL-1 in the NM group was significantly higher than in the normal diet group. These results suggested that NM promoted mRNA expression of genes involved in osteoblastogenesis in vivo. The increased protein expression of BMP2 (28.20-fold) and Wnt3a (4.17-fold) in the NM group was significantly higher than in the low-Ca group. In addition, the expression of BMP-2 increased by 3.57-fold in the NM group compared to the normal diet group. The protein expression of p-SMAD 1/5/8 and RUNX2 in the NM group increased by 3.32- and 3.76-fold, respectively, compared to the low-Ca group. These findings suggested that BMP-2-dependent p-SMAD 1/5/8/RUNX2/osteocalcin/COL-1 signaling was activated in OVX rats exposed to NM, which induced osteoblast differentiation.

### 3.12. NM Promotes in Vivo Osteoclastogenesis Related with the mRNA Expression of Genes

We investigated the changes in mRNA expression level of cathepsin K, TRAP, and RANK in the OVX rat model. As shown in Figure 7C, the mRNA expression of cathepsin K, TRAP, and RANK in the low-Ca group were significantly increased compared to that in sham group. In the NM diet group, the TRAP mRNA expression was dramatically decreased (24.63-fold) compared to the low-Ca group. In addition, cathepsin K mRNA expression level in the NM group was significantly reduced (15.62-fold) compared to that in low-Ca group. Furthermore, the level of RANK mRNA expression was 3.45-fold lower in the NM group than in the low-Ca group. In particular, the cathepsin K, TRAP, and RANK mRNA expression levels in the NM group were downregulated compared to the normal diet group. These results suggested that the NM diet reduced the expression of genes involved in osteoclastogenesis.

### 3.13. NM Promotes in Vivo Expression of Phosphorylated Mitogen-Activated Protein Kinases (MAPKs) JNK, ERK, and P38

Mitogen-activated protein kinase (MAPK) signaling is important for the regulation of osteoblast differentiation and maturation [44]. In Figure 7D, we evaluated the effects of the NM diet on the activation of MAPK signaling, including the expression levels of p-p38, p-JNK, and p-ERK for the regulation of osteoblast differentiation. The activation of p-p38, p-JNK, and p-ERK in the low-Ca group was significantly decreased compared to that in the sham group. However, the p-p38, p-JNK, and p-ERK levels in the NM diet group increased by 4.08-, 3.57-, and 3.68-fold, respectively, compared to the low-Ca group. Also, p-ERK and p-JNK expression increased in the NM group (1.26- and 1.32-fold, respectively) compared the normal diet group. Thus, our results suggest that the NM diet activate osteoblast differentiation and suppress osteoclast differentiation via the activation of MAPK signaling pathways.

## 4. Discussion

Recently, several studies have showed intrinsic bone formation effect of certain clay in vitro in the bone marrow and adipose-derived stromal cell cultures, when dispersed in biomaterials or applied directly to cells as a culture supplement [45]. Our studies appreciated whether NM inhibits osteoporosis in the OVX rats. The present study is reported the first time that an NM suspension enhanced osteoblast proliferation, mineralization, and osteogenic related gene. Also, our results suggest that NM shows novel biomaterial properties, with optimal chemical composition, and regulates bone formation or resorption by modulating osteoblasts and osteoclasts during osteoporosis.

NM is composed of 3.66 wt% Ca and has a size of 179 nm in diameter. The NM-supplemented group showed a 33.7% increase in the tibial Ca content, compared to rats in the low-Ca group. Moreover, the breaking force required in the NM group was 33.2% higher than in the low-Ca group. In addition, these results agree with those reported by Nordin et al., who showed that OVX rats had increased renal Ca loss; this could contribute to OVX-induced osteoporosis [46]. Our study showed that NM treatment of OVX rats fed a low-Ca diet decreased induction of osteoporosis. Generally, bone strength reflects bone quality. Bone quality parameters include bone microarchitecture, damage accumulation, and bone mineralization [47]. In our study, the NM suspension significantly interacted with the bone metabolism. These results showed that there is a possibility that the effects of NM were mainly mediated by supplementation of a natural Ca-type NM.

Our micro-CT results showed that the upper and lower cross-sections of the tibia had few trabeculae in the marrow region in the low-Ca group. However, in similar sections from the sham and NM treated groups, the bone marrow space was filled with relatively uniform plate-like trabecular structures that formed a well-connected bone structure (Figure 5A). The observed increase in BMD, cortical BMD, BV/TV, and Ct.Th, and the suppression in Tb.N and Tb.Sp, showed an increased overall bone strength and tibial trabecular and cortical bone density. The effects of OVX on bone are smaller in the cortical compartments than in the trabecular compartments [48]. Several reports have shown a reduction in BV/TV, Tb.Th, and Ct.Th and enhancement in BSA/BV, Tb.N, and Tb.Sp following OVX [34]. Our present findings in OVX rats are consistent with these observations. Moreover, we observed that NM affected the trabecular microarchitecture. Taken together, the micro-CT analysis indicated that NM attenuated OVX-induced osteoporosis. Bone regeneration is related to an induction of osteoblast cell proliferation, ALP activation, intracellular Ca deposition, mineralization and extracellular matrix formation [49,50,51]. As shown in Figure 2A, 250–1000 μg/mL of NM increased ALP activity in a dose dependent manner. Similar observations have been reported using osteoblasts grown on laponite nanoparticle gels, akermanite bioceramics, and titania; improvements in bone differentiation markers have also been reported, including accelerated mineralization and increased ALP activity in the bone matrix [49,52,53].

The present study showed that NM treated group could increase ALP activity and gene expression of the osteogenic makers RUNX2, osteocalcin, BMP-2, COL-1, Wnt3a, and p-SMAD 1/5/8. These osteoblast-related markers are participated in bone regeneration, formation, and metabolism [51,54,55]. In addition, a previous study on the effects of genistein on osteoblast-associated metabolism proved that genes related to the BMP/SMAD signaling pathway showed the strongest enhancements in human bone marrow stromal cell (hBMSC) cultures [53]. This is similar to our results, and these findings prove that BMP/SMAD signaling is an important regulator of hBMSC differentiation into an osteoblast lineage, with BMP2-dependent SMAD5 signaling as a downstream pathway [55]. Also, the expression level of RUNX2 and its downstream gene osteocalcin increase in hBMSCs, indicating an important role of hBMCs in osteoblast differentiation [55,56]. In addition, recent studies reported that canonical Wnt signaling contributed to bone formation through enhancement of the RUNX2 transcription factor that drives osteoblast differentiation [57]. Consistent with these previous observations, our findings proved that BMP/RUNX2/SMAD signaling was of considerable importance for the enhancement of the osteoblast lineage observed in the presence of NM.

Osteoclasts use to pivotal role in the bone resorption related with osteoporosis [58]. RANKL binds to RANK on preosteoclasts, stimulating their differentiation into osteoclasts [59]. Additionally, the RANKL-RANK interaction activates various osteoclastogenesis associated genes, such as cathepsin K and TRAP, via many downstream effectors [58]. Several studies showed that cathepsin K is an important osteoclast protease involved in bone matrix degradation [60]. Cathepsin K participates in osteoclast induced degradation of the sub-osteoclastic collagenous bone matrix [60,61,62]. The osteoclast enzyme TRAP is a substrate for cathepsin K and has been used as a molecular marker for osteoclasts [61,63]. Therefore, the inhibition of osteoclast formation may be a key mechanism for osteoporosis therapeutic agents. Interestingly, our results from TRAP staining and RT-PCR analysis of the gene expression level of cathepsin K, TRAP and RANK observed that NM significantly suppressed osteoclast formation.

Bone remodeling is influenced by many environmental factors and signaling pathways [64]. Previous studies have showed that the MAPK signaling pathway, such as p38, JNK, and ERK, is pivotal for the regulation of osteoblast differentiation, cell proliferation, and bone skeletal development [65]. Yoo et al. demonstrated that 1% Ca supplementation activates osteoblasts and reduces osteoclasts via the ERK and JNK pathways [34]. Also, Xio et al. reported that the effect of akermanite on BMSCs derived from OVX rats (BMSC-OVX) might be related to the MAPK signaling pathway [49]. In another study, Lin K et al. reported that substituted calcium silicate-bioactive ceramics upregulated ERK and p38 in BMSC-OVX [66]. We showed that the activation of p38 significantly upregulated in the NM treated group compared to the low-Ca group, both in vitro and in vivo. Thus, our study suggests that NM activates BMP-2/ RUNX2/p-SMAD 1/5/8 induced osteoblast differentiation and inhibits TRAP/RANK-induced osteoclastogenesis via the p38 pathway in the OVX rat model.

## 5. Conclusions

**Figure 8:**
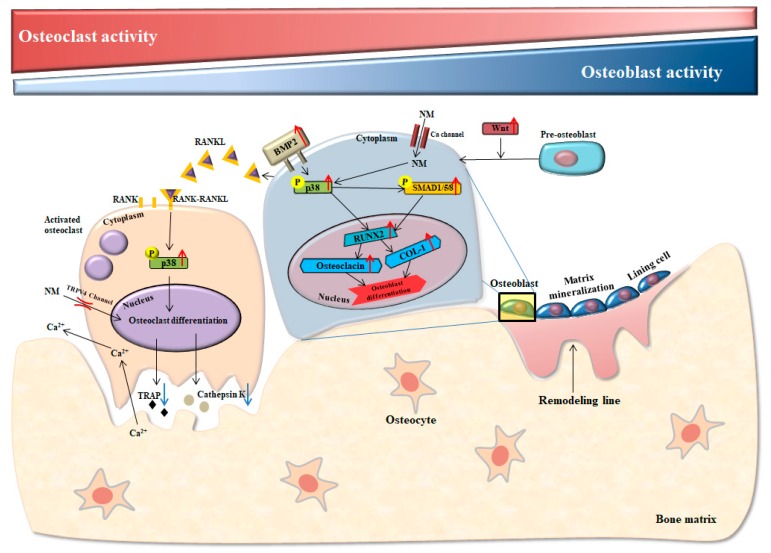
Effect of nano-montmorillonite on osteoblastogenesis and osteoclastogenesis.

For the first time, our study observed that natural Ca-type NM enhanced osteoblast proliferation, osteogenic differentiation, mineralization, and expression of osteoblast differentiation genes. NM improved proliferation, ALP activity, mineralization, and expression of osteoblast differentiation markers (BMP-2, p-SMAD 1/5/8, RUNX2, Wnt3a, osteocalcin, and COL-1) in an osteoblastic cell line in vitro and in a model of osteoporosis in vivo. In addition, our results from TRAP staining of the osteoclast differentiation markers TRAP, cathepsin K, and RANK showed that NM could significantly suppress osteoclast formation (Figure 8). The findings of our study suggest that the anti-osteoporosis effects of NM on the induction of osteoblasts and reduction in osteoclast formation and may help to explain the important role of clay for the treatment of bone metabolism disorders.

## Supplementary Materials

The following are available online at https://www.mdpi.com/2079-4991/10/2/230/s1: Table S1: Bodyweight and food intake. Table S2: Primers used for RT-PCR analysis. Table S3: Composition of the experimental diets. Figure S1: In vivo biochemical analyses.
