# Supplementary material for: Effect of Nano-Montmorillonite on Osteoblast Differentiation, Mineral Density, and Osteoclast Differentiation in Bone Formation
Source: Nanomaterials (Basel). 2020 Jan 28;10(2):230. doi: 10.3390/nano10020230 (PMC7075198; doi:10.3390/nano10020230)
Supplement: Supplementary file 1 [file nanomaterials-10-00230-s001.pdf]

Supplementary data

Effect of Nano-Montmorillonite on Osteoblast Differentiation, Mineral Density, and Osteoclast Differentiation in Bone Formation

Gyeong-Ji Kim, Daniel Kim, Kwon-Jai Lee, Daeyoung Kim Kang-Hyun Chung, Jeong Woo Choi, and Jeung Hee An**

Table S1. Bodyweight and food intake.

	Sham	Low Ca	Normal Diet	NM[a]
Feed intake (g/day)	16.78 ± 2.09[a]	17.62 ± 1.82[a]	16.75 ± 1.82[a]	16.59 ± 0.36[a]
Body weight (g)				
Initial	117.33 ± 8.41[a]	118.75 ± 7.68[a]	118.50 ± 6.95[a]	119 ± 6.89[a]
Final	339.75 ± 16.34[a]	324.25 ± 30.89[a]	338.25 ± 32.34[a]	344.25 ± 16.19[a]
Body weight gain (g/week)	27.8 ± 4.2[a]	25.68 ± 3.6[a]	27.46 ± 3.62[a]	27.46 ± 3.62[a]
FER[b]	0.23 ± 0.02[a]	0.2 ± 0.01[a]	0.23 ± 0.01[a]	0.23 ± 0.01[a]

[a] **NM:** Nano-Montmorillonite

[b] **FER:** Feed efficiency ratio = body weight gain (g/week)/feed intake (g/week)

Table S2. Primers used for RT-PCR analysis.

Target Gene	Species	Primer Sequence (5' → 3')	Annealing Temperature
**GAPDH**	**Human**	**F: GATGGTACATGACAAGGTGC**<br>**R: CAAGAAGGTGGTGAAGCAGG**	**60°C**
**BMP-2**	**Human**	**F: TCATAAAACCTGCAACAGCCAACTCG**<br>**R: GCTGTACTAGCGACACCCAC**	**60°C**
**RUNX2**	**Human**	**F: CCGCACGACAACCGCACCAT**<br>**R: CGCTCCGGCCCACAAATCTC**	**60°C**
**Osteocalcin**	**Human**	**F: GGCCAGGCAGGTGCGAAGC**<br>**R: GCCAGGCCAGCAGAGCGACAC**	**65°C**
**COL-1**	**Human**	**F: AGCGCTGGTTTCGACTTCAGCTTCC**<br>**R: CATCGGCAGGGTCGGAGCCCT**	**60°C**
**GAPDH**	**Mouse**	**F: ACTTTGTCAAGCTCATTTCC**<br>**R: TGCAGCGAACTTTATTGATG**	**58°C**
**RANK**	**Mouse**	**F: AAGATGGTTCCAGAAGACGGT**<br>**R: CATAGAGTCAGTGCTCGGA**	**63°C**
**TRAP**	**Mouse**	**F: ACTTCCCCAGCCCTTACTACCG**<br>**R: TCAGCACATAGCCCACACCG**	**63°C**
**Cathepsin K**	**Mouse**	**F: CTGAAGATGCTTTCCCATATGTGGG**<br>**R: GCAGGCGTTGTTCTTATTCCGAGC**	**63°C**
**GAPDH**	**Rat**	**F: AACTCCATTCCACCTT**<br>**R: GAGGGCCTCTCTCTTGCTCT**	**48°C**
**BMP-2**	**Rat**	**F: AAGGCACCCTTTGTATGTGGACT**<br>**R: CATGCCTTAGGGATTTTGGA**	**55°C**
**RUNX2**	**Rat**	**F: TCCAGCCACCTTCACTTACAC**<br>**R: GCGTCAACACCATCATTCTG**	**53°C**
**Osteocalcin**	**Rat**	**F: AGCTCAACCCCAATTGTGAC**<br>**R: AGCTGTGCCGTCCATACTTT**	**55°C**
**COL-1**	**Rat**	**F: TTGACCCTAACCAAGGATGC**<br>**R: CACCCCTTCTGCGTTGTATT**	**57°C**
**Wnt3a**	**Rat**	**F: TCCGACTCTTGGCAGAACTT**<br>**R: AATGGAATAGGTCCCGAACA**	**51°C**
**RANK**	**Rat**	**F: GTGACTCTCCAGGTCACTCC**<br>**R: GGCAGACACACACTGTCG**	**60°C**
**TRAP**	**Rat**	**F: CGCCAGAACCGTG CAGA**<br>**R: TCAGGCTGCTGGCTGAC**	**60°C**
**Cathepsin K**	**Rat**	**F: CCCGAGCTCCATCGACTATCG**<br>**R: CTGTACCCTCTGCACTTAGCTGCC**	**60°C**

Table S3. Composition of the experimental diets.

Composition	Sham	Low Ca	Normal Diet	NM[a]
**Casein (g/kg)**	**200.0**	**200.0**	**200.0**	**200.0**
**L-Cystine (g/kg)**	**3.0**	**3.0**	**3.0**	**3.0**
**Sucrose (g/kg)**	**334.288**	**342.188**	**334.288**	**342.188**
**Corn Starch (g/kg)**	**313.0**	**320.0**	**313.0**	**320.0**
**Soybean Oil (g/kg)**	**60.0**	**60.0**	**60.0**	**60.0**
**Cellulose (g/kg)**	**40.0**	**40.0**	**40.0**	**40.0**
**Mineral Mix, (g/kg)[b]**	**13.37**	**13.37**	**13.37**	**13.37**
**Potassium Phosphate, Monobasic (g/kg)**	**11.43**	**11.43**	**11.43**	**11.43**
**Vitamin Mix, (g/kg)[c]**	**10.0**	**10.0**	**10.0**	**10.0**
**Calcium (%)**	**0.6**	**0.01**	**0.6**	**0.01**
**P (%)[d]**	**0.4**	**0.4**	**0.4**	**0.4**
**NM (mg/kg)**	**-**	**-**	**-**	**50**

[a] **NM :** Nano-Montmorillonite

[b] **Mineral Mix (g/kg) :** (NaCl: 193.7325, $C_6H_7K_3O_8$: 575.9615, K_2SO_4: 136.1363, MgO: 62.8322, $MnCO_3$: 9.163, $C_6H_5FeO_7$: 15.708, $ZnCO_3$: 4.1888, $CuCO_3$: 0.7854, KIO_3: 0.0262, $Na_2SeO_3{\cdot}5H_2O$: 0.0262, $CrK(SO_4)_2{\cdot}12H_2O$: 1.4399).

[c] **Vitamin Mix (g/kg) :** (*p*-Aminobenzoic Acid: 11.0132, Vitamin C, Ascorbic Acid, Coated (97.5%): 101.6604, Biotin: 0.0441, Vitamin B_{12} (0.1% in mannitol): 2.9736, Calcium Pantothenate: 6.6079, Choline Dihydrogen Citrate: 349.6916, Folic Acid: 0.1982, Inositol: 11.0132, Vitamin K_3, Menadione: 4.9559, Niacin: 9.9119, Pyridoxine HCl: 2.2026, Riboflavin: 2.2026, Thiamin (81%): 2.2026, Vitamin A Palmitate (500,000 IU/g): 3.9648, Vitamin D_3, Cholecalciferol (500,000 IU/g): 0.4405, Vitamin E, DL-Alpha Tocopheryl Acetate (500 IU/g): 24.2291, Corn Starch: 466.6878).

[d] **P :** Phosphorus

Supplementary Figure Legends

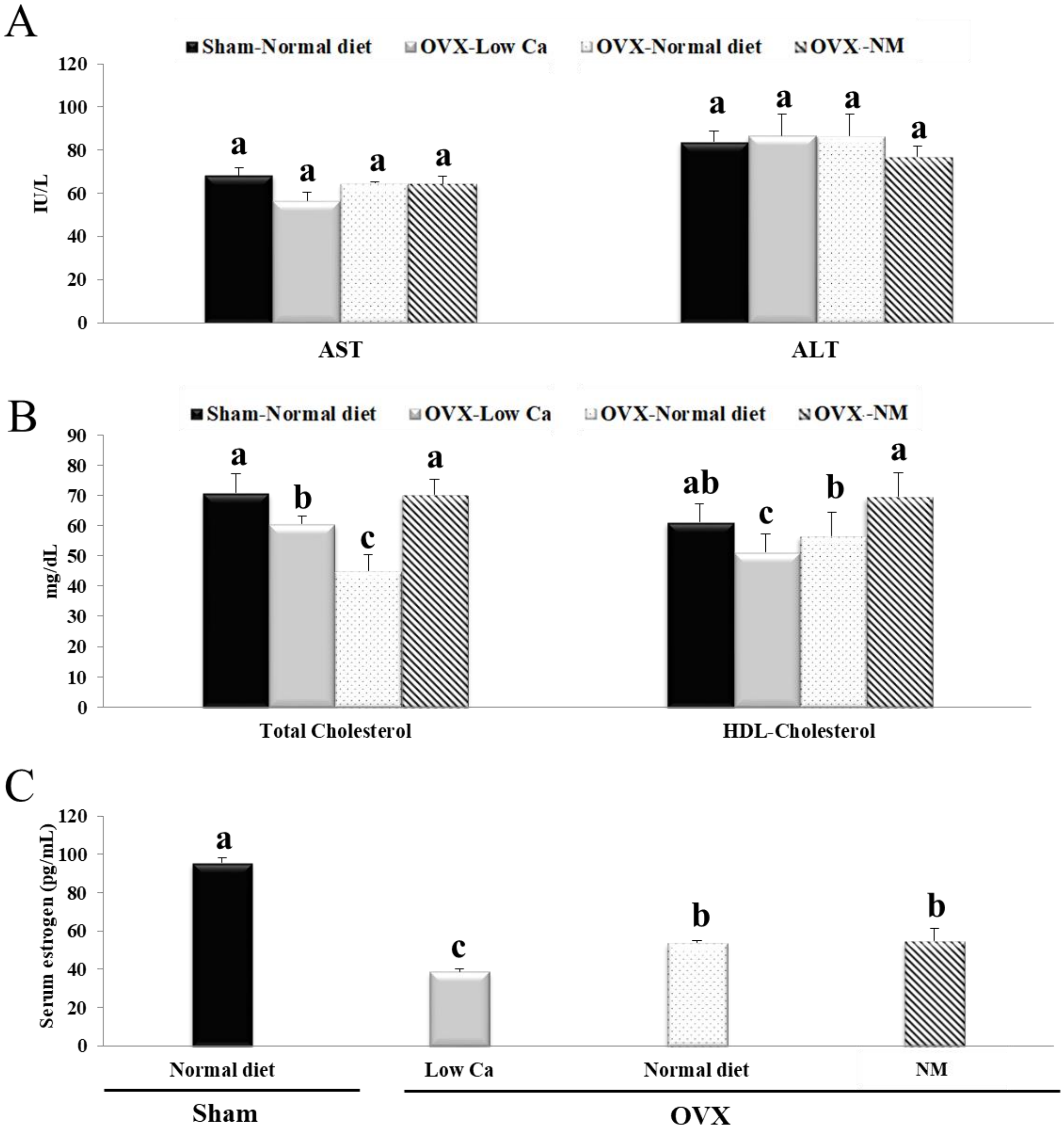

Figure S1. *In vivo* biochemical analyses of sham and ovariectomized (OVX) rats. Serum levels were determined for (A) alanine aminotransferase (ALT) and aspartate aminotransferase (AST); (B) total cholesterol and high-density lipoprotein (HDL-Cholesterol); and (C) estrogen. Values represent the mean ± SD (n = 3). Values not sharing a common superscript (a-c) differed significantly (Duncan's multiple range test) ($p < 0.05$).
